# Midpalatal Suture: Single-Cell RNA-Seq Reveals Intramembrane Ossification and *Piezo2* Chondrogenic Mesenchymal Cell Involvement

**DOI:** 10.3390/cells11223585

**Published:** 2022-11-12

**Authors:** Lu Gao, Tiansong Xu, Liqi Zhang, Yuchen Li, Tianxing Yan, Guoxia Yu, Feng Chen

**Affiliations:** 1 Central Laboratory, National Clinical Research Center for Oral Diseases, Peking University School and Hospital of Stomatology, Beijing 100081, China; 2 Department of Stomatology, Beijing Children’s Hospital, Capital Medical University, National Center for Children’s Health, Beijing 100045, China

**Keywords:** midpalatal suture, single-cell RNA-sequencing, intramembrane ossification, *Piezo2* chondrogenic mesenchymal cells, maturation and ossification

## Abstract

The midpalatal suture is mainly responsible for the growth and development of the maxillary and resistance to rapid maxillary expansion (RME). It is essential for clinical researchers to explore the intramembrane ossification and to elucidate the underlying mechanism of the maturation and ossification process of the midpalatal suture to help identify the optimum time and force of RME. However, mechanistic studies associated with the midpalatal suture are rare. The aim of this present study is to create an intramembrane osteogenesis model for the midpalatal suture region of mice. Interestingly, we discovered a type of chondrogenic mesenchymal cell expressing *Piezo2*, which might be related to the detection of mechanical and external stimuli. This result provides a potential molecular and cellular mechanism that explains why the midpalatal suture is not closed until adulthood. We depict a landscape of mesenchymal cells that might play an important role in the intramembrane osteogenesis of the midpalatal suture and provide new perspectives on midpalate suture maturation and ossification, which might lead to further possibilities for clinical operations.

## 1. Introduction

Maxillary deficiency is a type of craniofacial malformation with high incidence (23.3%). Maxillary transverse deficiency plays an important role in maxillary deficiency and can lead to various malocclusions, including obstructive sleep apnea [1,2,3,4]. Dentofacial deformities including craniosynostosis and cleft lip/palate are accompanied by maxillary transverse deficiency [1,5]. Maxillary transverse deficiency impairs patients’ oral and maxillofacial development and function, facial aesthetics, and long-term health and quality of life [1,6].

Rapid maxillary expansion (RME), the routine treatment procedure to correct maxillary transverse deficiency, can involve tooth-borne expansion, micro-implant assisted expansion, and surgically assisted expansion [1,7]. The timing and treatment-induced trauma of various RME methods are distinctly different. Treatment timing is vital for determining the curative effects and severity of the side effects of each RME method [8]. Expansion with inappropriate timing can cause unnecessary trauma [8] and increase the severity of side effects [9,10,11,12]. The accurate appraisal of the maxillary transverse developmental status is critical when deciding on the appropriate timing for different treatment methods [1].

The midpalatal suture is the main site of maxillary growth and development and resistance to RME [13]. The accurate and efficient appraisal of the midpalatal suture maturation and ossification status is the basis for the appraisal of the maxillary transverse developmental status.

The midpalatal suture is known for its narrow and complex anatomical morphological characteristics [1]. Several appraisal methods for midpalatal suture maturation and ossification status have been reported, including histological methods and imaging methods. Histological evidence shows that the midpalatal suture may not be fully closed until late adolescence or even early adulthood [14,15,16,17,18]. Imaging evidence, especially from cone-beam computed tomography (CBCT) results, show that the maturation and ossification status of a midpalatal suture undergoes significant changes during the fast growth and development period, yet individual differences are obvious, indicating the necessity of further evidence regarding the midpalatal suture maturation and ossification process [19,20,21,22].

Single-cell RNA sequencing is a high-throughput sequencing technology at the single-cell level. Traditional bulk cell sequencing tests the total average response of the genetic information of cell populations or the genetic information of dominant cells. Therefore, it cannot reflect the real situation of each cell, and a particular cell’s function is unclear. Single-cell RNA sequencing is a technology for sequencing the transcriptome of a single cell that can help accurately measure the gene structure and expression status, reveal the differences between single cells, and analyze the heterogeneity of cells with the same phenotype [23]. Single-cell RNA sequencing is of great clinical value in research on malignant tumors, immunology, neuroscience, and embryonic development [24,25,26,27]. At present, most studies on the development of the midpalatal suture are based on histological methods, focusing on osteoblasts, osteoclasts, and chondrocytes [28,29,30]. There are no single-cell RNA sequencing results from studies on the maturation and ossification process of the midpalatal suture.

Mice and humans have high homology in terms of the process of craniomaxillofacial growth and development. Li et al. studied the effect of genes on bone remodeling in the midpalatal suture via a mouse model [31]. Koehne et al. determined the role of bone restitution in stress-mediated sutural bone growth via a mouse model [28]. Therefore, mouse models are suitable for the single-cell RNA sequencing of the maturation and ossification processes of the midpalatal suture.

In this study, we establish an intramembrane osteogenesis model of the midpalatal suture region. We discovered a type of chondrogenic mesenchymal cell expressing *Piezo2*, which will help us to identify the molecular and cellular mechanisms that explain why the midpalatal suture is not closed until adulthood.

## 2. Materials and Methods

### 2.1. Isolation of Cells from the Midpalatal Suture Region for Single-Cell RNA Sequencing

The experiments in this study were carried out in compliance with the ARRIVE guidelines and were approved by the Beijing Municipal Science and Technology Commission (MDKN-2022-011). Twenty-seven healthy specific pathogen-free (SPF) female C57BL6/J mice at 4, 5, and 9 weeks of age (nine mice at each age) were selected (Beijing Vital River Laboratory Animal Technology Co., Beijing, China). The animals were sacrificed by cervical dislocation, the skin and mandible were removed, and the cranial–maxillary complex was extracted. Then, nine mice (three at 4 weeks of age, three at 5 weeks of age, and three at 9 weeks of age) were used for cell isolation. The cranial–maxillary complex of each sample was split through the midpalatal suture by a blade and tissue cells on bilateral bone surfaces inside the midpalatal suture were scraped off and put into a preserving solution (Singleron GEXSCOPE, Jiangsu, China) and prepared for single-cell RNA sequencing analysis.

### 2.2. Histological Staining, Immunohistochemistry, and Micro-CT Analysis

After the animals were sacrificed, the other eighteen mice were divided evenly into two groups (three at 4 weeks of age, three at 5 weeks of age, and three at 9 weeks of age in each group). One group was used for staining analysis. The cranial–maxillary complex samples used for histological staining and immunohistochemistry analysis were immediately fixed in paraformaldehyde (PFA) 4% for 24 h, decalcified for 4 weeks, then dehydrated, embedded in paraffin, and serially sectioned at a 5 µm thickness.

For the anterior and the posterior part of the midpalatal suture position, hematoxylin-eosin (HE) staining, Masson staining, Sirius red (SR) staining, Van Gieson’s (VG) staining, and Safranin O-fast green (SOFG) staining were carried out. A further immunohistochemical analysis used *Piezo2* polyclonal antibody (26205-1-AP, Proteintech Group, Rosemont, IL, USA). All results were panoramically scanned under a light microscope for observation.

### 2.3. Micro-CT Analysis

After the animals were sacrificed, the nine mice used for the Micro-CT analysis were fixed in PFA 4% and immediately scanned using a vivaCT 80 (SCANCO, Zurich, Switzerland) at 10 µm resolution. The observations and measurements were conducted out using Inveon research workplace 4.2 (Siemens AG, Berlin and Munich, Germany). Linear distances and bone mass-related analyses were measured and calculated.

### 2.4. Single-Cell RNA Sequencing (scRNA-Seq) with Singleron GECSCOPE Platform

The midpalatal suture region cell populations were scratched off from each cranial–maxillary complex sample and collected into a preservation solution in sealed tubes. The concentration of the single-cell preparations was assessed using a hemocytometer in an inverted microscope and was adjusted to 1 × 10^5^ cells/mL in PBS (HyClone, Cytiva, Marlborough, MA, USA). Single-cell suspensions were loaded onto microfluidic devices. Then, scRNA-seq libraries were constructed on the basis of the Singleron GEXSCOPE^®^ protocol with GEXSCOPE^®^ Single-Cell RNA Library Kits (Singleron Biotechnologies). Individual libraries were diluted to 4 nM, and the pooled libraries were sequenced on an Illumina novaseq 6000 with 150 bp paired-end reads.

### 2.5. Single-Cell RNA Data Sequencing

A CeleScope (https://github.com/singleron-RD/CeleScope) v1.9.0 (Singleron Biotechnologies, Nanjing, China) pipeline was used to generate raw data, remove low quality reads, map reads to the reference genome GRCm38 (Ensembl version 92 annotation), extract cell barcodes and unique molecular identifiers (UMIs), and obtain expression matrix files for subsequent analyses. R package Seurat (version 4.0.2, CRAN, New York, NY, USA) (https://github.com/satijalab/seurat) was employed to perform quality control, normalization, and downstream analyses. Then, the UMI matrix was processed to preserve high-quality cells that expressed 200 to 5000 genes and those with less than 15% mitochondrial genes. Data integration was carried out with ‘FindIntegrationAnchors’ and ‘IntegrateData’. A regression was performed before clustering with ‘CellCycleScoring’ to reduce cell cycle heterogeneity.

### 2.6. Dimensionality Reduction, Cell Clustering, and Identification for scRNA-Seq Data

Dimension reduction and unsupervised clustering were carried out following the standard workflow of Seurat. The first 50 PCs and a resolution of 0.2 were applied to cluster the data into cell types. Each cell cluster was annotated manually according to the marker genes with ‘FindAllMarkers’, which identified the major cell types after the first round of unsupervised clustering. Then, we extracted mesenchymal cells and performed second-round clustering with the same workflow and parameters. R package was used for gene set over-representation analysis. The single-cell pseudotime trajectories were calculated with the Monocle3 R package (version.2.18.0, http://cole-trapnell-lab.github.io/monocle-release/, Cole Trapnell Lab, Washington, Seattle, WA, USA) and the veloctyo.py v0.17.16 (Kharchenko Lab, Harvard Medical School, Cambridge, MA, USA; Linnarsson Lab, Karolinska Institutet, Stockholm, Sweden) and scVelo algorithms (0.2.5.dev5 + g1805ab4 (python 3.8.0, Python, Wilmington, DE, USA).

### 2.7. Statistical Analysis

Statistical analyses were performed with Origin software (2020b, Origin, Irvine, CA, USA) and R software. Statistical significance was computed via a non-parametric Wilcoxon rank sum test and ANOVA analysis. *p* values of ≤0.05 were regarded as statistically significant.

## 3. Results

To explore the potential mechanism of the midpalatal suture maturation and ossification process, we established a mouse model that recapitulated the cellular and molecular composition of the midpalatal suture, combining it with classical histological staining methods and Micro-CT analyses. We characterized the cell populations and osteogenic processes in the midpalatal suture and then focused on the *Piezo2^+^* chondrogenic mesenchymal cell populations.

### 3.1. Histological Staining and Micro-CT Analysis of the Midpalatal Suture in the Mouse Model

**Figure 1:**
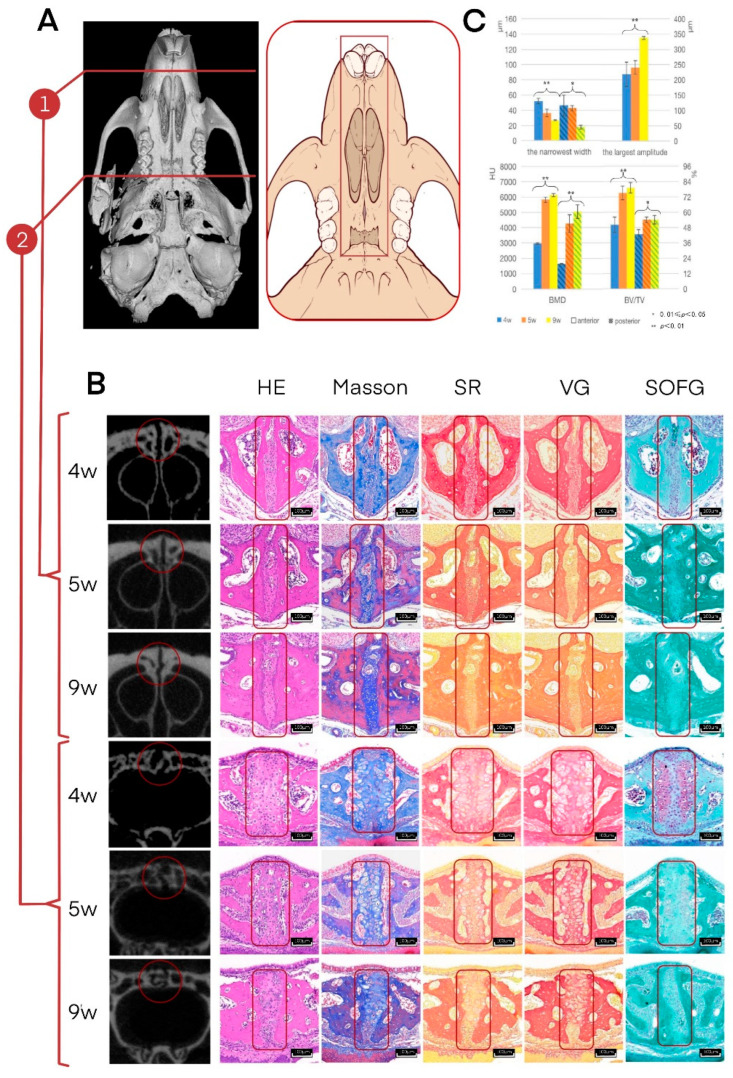
Histological staining and Micro-CT analysis of the midpalatal suture (mouse model). (**A**) Histological staining and Micro-CT analysis are shown at two typical sections: the anterior (the palatal plate part of the maxilla) and the posterior (the horizontal plate part of the palatal bone). (**B**) The Micro-CT coronal view, hematoxylin-eosin (HE) staining, Masson staining, Sirius red (SR) staining, Van Gieson’s (VG) staining, and Safranin O-fast green (SOFG) staining are shown in order, at 4, 5, and 9 weeks of age. Scale for Micro-CT section image: 40×, scale for histological staining: 12×. (**C**) Micro-CT statistical results are shown in bar charts, including the narrowest width, the largest amplitude, the bone mineral density (BMD), and the bone volume/tissue volume (BV/TV).

With the maturation process, the curvature of the midpalatal suture increased, and the bilateral maxilla or palatal bones became more interdigitated, especially in the posterior part. The shape of the midpalatal suture from the coronal view was mainly straight at 4 and 5 weeks of age and then became more curved at 9 weeks of age, which was also indicated by the significant increase in the largest amplitude from 4 weeks to 9 weeks of age (Figure 1). However, the midpalatal suture at 9 weeks of age in mice (equal to 16–17 years in humans) remained unclosed, which was also demonstrated by histological staining (Figure 1). In addition, the width of the midpalatal suture decreased, while the bone mineral density (BMD) and bone volume/tissue volume (BV/TV) obviously increased from 4 weeks to 9 weeks of age (Figure 1). The Micro-CT results suggest that during the maturation process, the midpalatal suture becomes more interdigitated in shape, narrower in width, and more ossified. However, it should be noted that the midpalatal suture remains unfused at 9 weeks of age (mouse model).

The histological staining observation verified the Micro-CT analysis results. The midpalatal suture region was mainly composed of fibrocytes, fibers, and osteoblasts, as is shown by the hematoxylin-eosin (HE) staining, Masson staining, Sirius red (SR) staining, and Van Gieson’s (VG) staining. Chondrocytes were observed at 4 weeks of age but were rarely seen at 5 and 9 weeks of age, as is shown by the Safranin O-fast green (SOFG) staining. The fibers were basically parallel and evenly arranged and were connected to the bilateral palatine plate of the maxilla or the horizontal plate of the palatine bone surface (Figure 1). During the maturation and ossification process, the morphological changes in the posterior part of the midpalatal suture (the horizontal plate part of the palatal bone) were more significant than in the anterior part (the palatal plate part of the maxilla).

### 3.2. Single-Cell RNA Sequencing Analysis of the Midpalatal Suture in the Mouse Model

**Figure 2:**
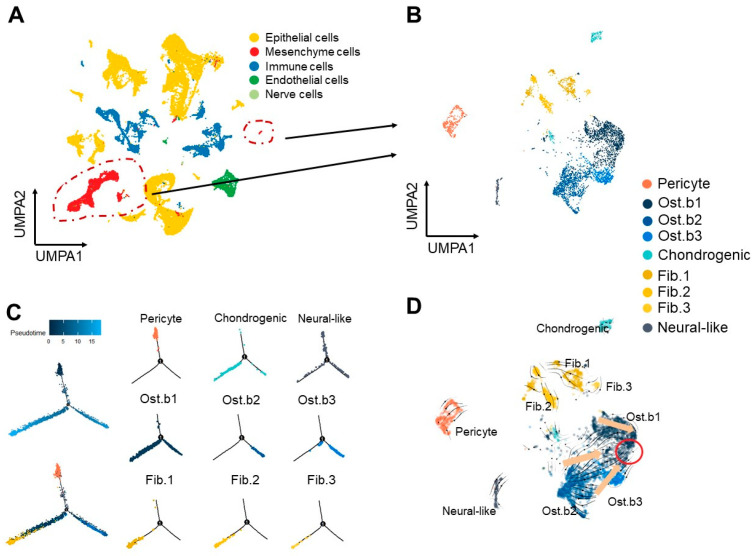
Mesenchymal cells show differentiation potential into osteoblasts, chondroblasts, and fibroblasts in the midpalatal suture (mouse model). (**A**) Distribution of five cell clusters in the midpalatal suture visualized using UMAP plots, including epithelial cells, mesenchyme cells, immune cells, endothelial cells, and nerve cells. The area circled by the dotted line shows mesenchyme cells magnified on the right in (**B**). (**B**) Distribution of 4678 re-clustered mesenchymal cells. Nine sub-clusters are visualized in total in the UMAP plots. (**C**) Pseudotime trajectory analysis showing the relationships between nine subclusters of mesenchymal cells colored by cell cluster. (**D**) RNA velocity of each cluster of mesenchymal cells calculated from unspliced and spliced transcripts colored by cell cluster.

To analyze the cellular and molecular composition of the midpalatal suture region with single-cell RNA sequencing, after quality control to remove low-quality cells, we found 45,677 cells in total, including 16,686 cells from 4-week-old mice, 17,053 cells from 5-week-old mice, and 11,938 cells from 9-week-old mice (Figure S1). First, the five major cell types were identified, including epithelial cells, mesenchyme cells, immune cells, endothelial cells, and nerve cells, which are shown by Uniform Manifold Approximation and Projection for Dimension Reduction (UMAP) (Figure 2A and Table S1). Mesenchymal cells were selected from the whole cell populations due to their essential role in the maturation and ossification process, especially bone matrix formation.

Through unsupervised clustering and marker analysis, we found that mesenchymal cells consisted of five distinct clusters according to marker gene expression, including pericytes (*Acta2*, *Rgs5*, and *Nes*), osteoblastic mesenchymal cells (*Ibsp*, *Mmp13*, and *Bglap2*), chondrogenic mesenchymal cells (*Col2a1*, *Col9a1*, *Col9a2*, and *Ucma*), fibroblastic mesenchymal cells (*Smoc2*, *Col8a1*, and *Dpep1*), and neural-like cells (*Olig2*, *Rtn1*, and *Elavl4*) from mesenchymal stem cells (MSCs) (Figure 2B and Figures S2 and S3). The cell populations are shown separately for each age group in Figure S4 and Table S3.

Pseudotime and RNA velocity analyses displayed a differentiation trajectory in each cluster of mesenchymal cells and further demonstrated that the nine clusters were in different states. In the pseudotime analyses, pericytes were located in the upper branch, osteoblastic mesenchymal cells-1 (Ost.b1) were in the two lower branches, osteoblastic mesenchymal cells-2 (Ost.b2) and osteoblastic mesenchymal cells-3 (Ost.b3) were in the lower right branch, and the fibroblastic mesenchymal cells-1,2,3 (Fib.1, Fib.2, Fib.3) were in the lower left branch (Figure 2C and Figure S5).

RNA velocity analyses suggested that although the three osteoblastic mesenchymal cell clusters had different situations, they differentiated towards the same destination (Figure 2D).

### 3.3. The Function of Different Clusters of Osteoblastic Mesenchymal Cells in Intramembrane Osteogenesis of the Midpalatal Suture

To depict the osteogenic process of the midpalatal suture, we performed differentially expressed gene (DEGs) and pathway analyses.

**Figure 3:**
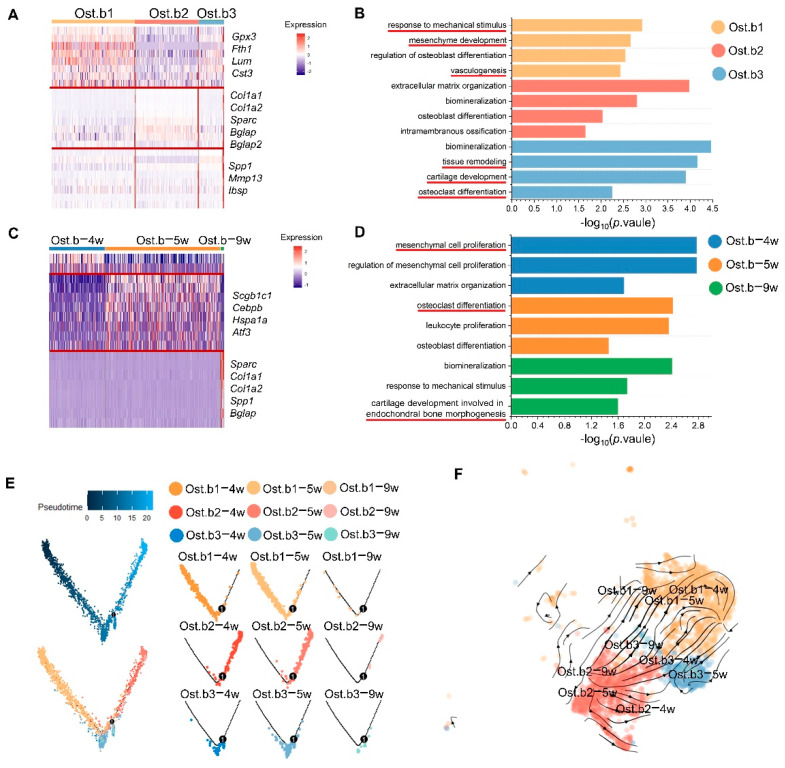
Different clusters of osteoblastic mesenchymal cells have different roles in the intramembrane osteogenesis of the midpalatal suture (mouse model). (**A**,**C**) Heatmaps showing the average expression of the top differentially expressed genes for each cluster of osteoblastic mesenchymal cells divided by unsupervised clustering ((**A**), Ost.b1, Ost.b2, and Ost.b3) and over time (**C**), Ost.b−4w, Ost.b−5w, and Ost.b−9w). (**B**,**D**) Bar graphs showing the enriched gene ontology terms between Ost.b1, Ost.b2, and Ost.b3 (**B**) and between Ost.b−4w, Ost.b−5w, and Ost.b−9w (**D**). (**E**) Pseudotime trajectory analysis of nine subclusters of osteoblastic mesenchymal cells colored by cell cluster. (**F**) RNA velocity of each cluster of osteoblastic mesenchymal cells colored by cell cluster.

In terms of the subclusters of mesenchymal cells, Ost.b1 upregulated pathways related to mesenchyme development and vasculogenesis; Ost.b2 were associated with biomineralization and extracellular matrix organization; and the genes associated with tissue remodeling, cartilage development, and osteoclast differentiation were upregulated in Ost.b3 (Figure 3A,B and Table S4).

In terms of time classification, osteoblastic mesenchymal cells in 4-week-olds (Ost.b-4w) displayed a potential toward mesenchymal cell proliferation; mesenchymal cells in 5-week-olds (Ost.b-5w) upregulated pathways linked to osteoclast and osteoblast differentiation; and genes related to cartilage development involved in endochondral bone morphogenesis were upregulated in mesenchymal cells in 9-week-olds (Ost.b-9w) (Figure 3C,D and Table S5).

Furthermore, we examined the trajectory of the osteoblastic mesenchymal cells by time and subcluster. The results showed that Ost.b1 were mainly positioned in the left branch, Ost.b2 were mostly located in the right branch, and Ost.b3 were distributed in both the left and the right branches, suggesting that each osteoblastic subcluster differentiated in different states (Figure 3E). RNA velocity analyses indicated similar results (Figure 3F).

### 3.4. Identification of Piezo2^+^ Chondrogenic Mesenchymal Cells

**Figure 4:**
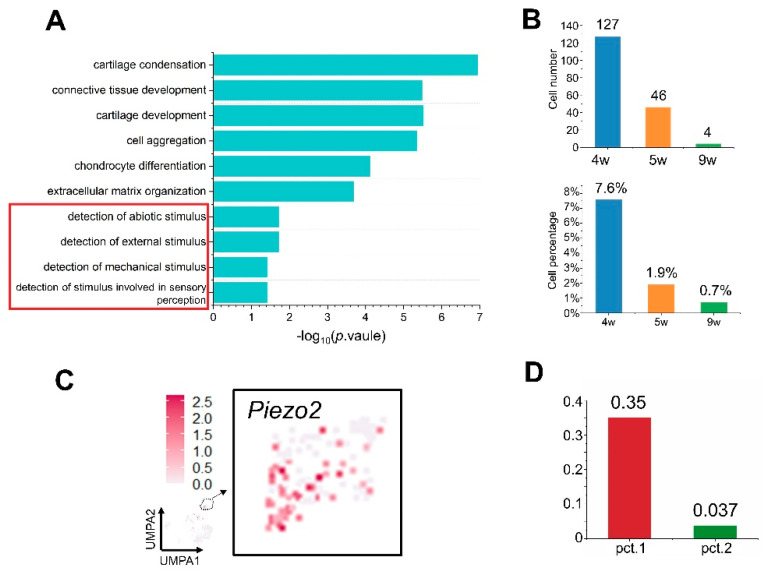
Identification of *Piezo2^+^* chondrogenic mesenchymal cells by scRNA-seq (mouse model). (**A**) Bar graph showing the enriched gene ontology terms of chondrogenic mesenchymal cells. (**B**) Bar graph showing the cell numbers and percentage of chondrogenic mesenchymal cells. (**C**) Feature plots showing the expression of *Piezo2^+^* mesenchymal cells. Additionally, feature plots of *Piezo2^+^* chondrogenic mesenchymal cells have been magnified and are visualized on the magnified UMAP and color coded by the expression level of *Piezo2*. (**D**) Bar graphs showing pct.1 and pct.2 of *Piezo2^+^* of chondrogenic mesenchymal cells calculated with DEGs.

Since chondrocytes play a role in intramembranous ossification [32], we studied chondrogenic mesenchymal cell identification in the midpalatal suture and analyzed the cell number, percentage, and pathway enrichment (Figure 4A,B). The upregulated pathways in these cells were related to cartilage condensation, connective tissue development, and the detection of mechanical stimuli. Tensional force stimulation play an important role in the development and differentiation processes of osteochondral progenitor cells in the secondary cartilage [33]. We focused on the effect of mechanical stimuli on the midpalatal suture in order to explain why the midpalatal suture remains unclosed until adulthood. Several known genes associated with mechanical stimuli were selected to explore the possible mechanisms [34,35,36,37] (Figure S6).

Interestingly, *Piezo2*, a classic marker gene associated with mechanosensation ion channels, was calculated via DEGs by comparing chondrogenic mesenchymal cells to other mesenchymal cells (Log_2_FC  =  0.917, *p*  =  6.57 × 10^−74^) (Figure 4C). There is a great difference in the expression multiples, which is close to 10 times, for pct.1 (0.35) and pct.2 (0.037), indicating that *Piezo2* is a specific gene for chondrogenic mesenchymal cell clusters (Figure 4D).

### 3.5. The Potential Role of Piezo2^+^ Chondrogenic Mesenchymal Cells Might Be Linked to the Detection of Mechanical Stimuli and Wound Healing

**Figure 5:**
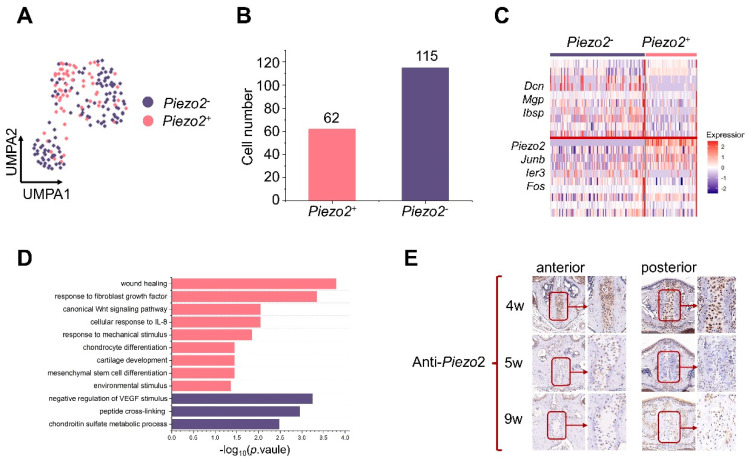
*Piezo2^+^* chondrogenic mesenchymal cells are linked to the detection of mechanical stimuli. (**A**) Feature plots showing the expression of *Piezo2^+^* and *Piezo2*^−^ chondrogenic mesenchymal cells color coded by whether the cells expressed *Piezo2* or not. (**B**) Bar graphs showing the cell number of *Piezo2^+^* (62 cells) and *Piezo2*^−^ (115 cells) chondrogenic mesenchymal cells. (**C**) Heatmap showing the average expression of the top differentially expressed genes for *Piezo2^+^* and *Piezo2*^−^ chondrogenic mesenchymal cells. (**D**) Bar graphs showing the enriched gene ontology terms of *Piezo2^+^* and *Piezo2*^−^ chondrogenic mesenchymal cells. (**E**) The immunohistochemical staining results of *Piezo 2* are shown in order at 4, 5, and 9 weeks of age. Scale: 12× and 24× (magnified on the right).

We distinguished the transcriptional differences between *Piezo2^+^* chondrogenic mesenchymal cells from *Piezo2*^−^ ones (Figure 5A,B). We performed DEGs and pathway enrichment analyses.

Compared to *Piezo2*^−^ ones, *Piezo2^+^* chondrogenic mesenchymal cells showed a higher expression of genes related to mechanical stimuli (*Junb*, *Fos*, and *lers*) and appeared to be associated with wound healing, response to fibroblast growth factor, mesenchymal stem cells, and chondrocyte differentiation (Figure 5C,D and Tables S6 and S7). We validated the presence of *Piezo2^+^* chondrogenic mesenchymal cells with immumohistochemical staining against *Piezo2*, and the results agreed well with the data above (Figure 5E).

Similarly, we also analyzed *Piezo2^+^* and *Piezo2*^−^ fibroblastic mesenchymal cells using the same method (Figure S7).

## 4. Discussion

In the present study, we described the intramembrane osteogenesis of the midpalatal suture and discovered a type of chondrogenic mesenchymal cell expressing *Piezo2* with single-cell sequencing, which helps clarify the maturation and ossification processes of the midpalatal suture and explain why the midpalatal suture is not closed until adulthood. Single-cell sequencing could distinguish the specific cell clusters, which cannot be identified by traditional staining techniques, and depict the molecular signatures of the mesenchymal cell populations.

First, osteoblastic mesenchymal cells are divided into three clusters that play different roles in intramembrane osteogenesis. Our osteogenesis model was in accord with the classical intramembrane ossification that occurs in flat bones, including the maxilla, mandible, and clavicle [38].

**Figure 6:**
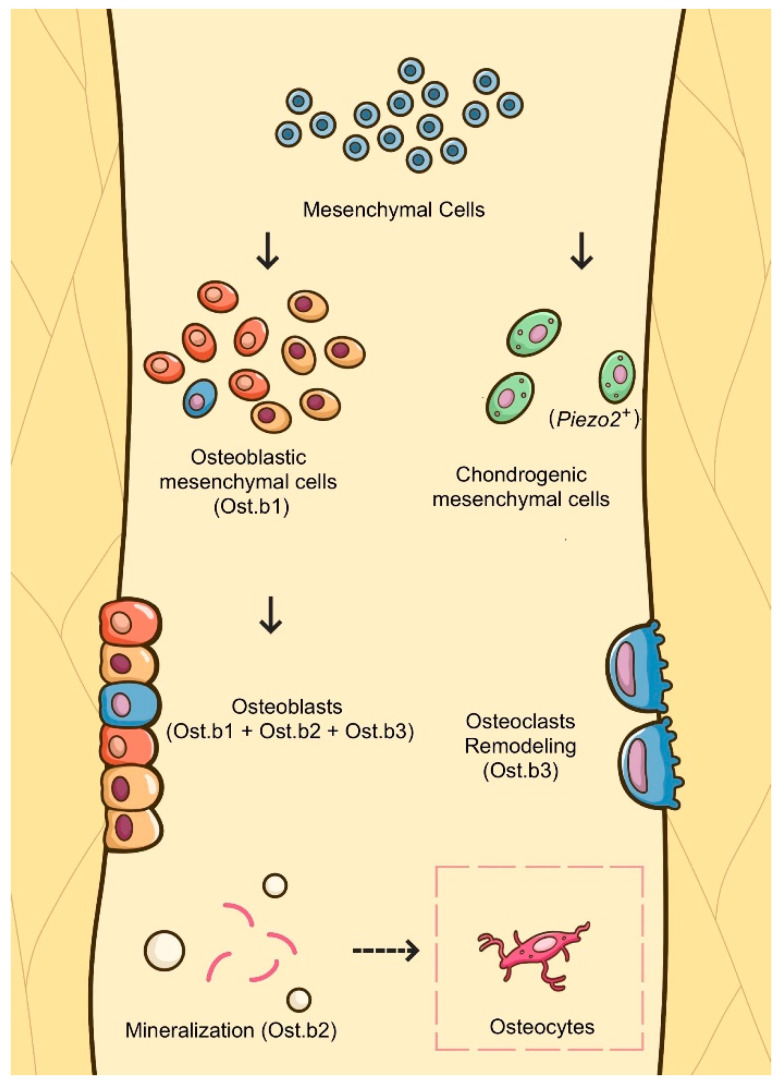
A schematic diagram recapitulating the mechanism in the maturation and ossification processes of the midpalatal suture. Mesenchymal cells are selected for their potential involvement in the maturation and ossification process of the midpalatal suture. Osteoblastic mesenchymal cells are divided into three clusters (Ost.b1, Ost.b2, and Ost.b3) and play various roles in intramembrane osteogenesis processes. Mesenchymal cells proliferate, condense (Ost.b1), and differentiate into osteoblasts (Ost.b1, Ost.b2, and Ost.b3). Osteoblasts shape an ossification center, secrete osteoid, induce mineralization (Ost.b2), and transform into osteocytes. Bone remodeling emerges throughout the whole procedure (Ost.b3). Additionally, *Piezo2^+^* chondrogenic mesenchymal cells might be linked to mechanical stimulus detection and may play a key role in the osteogenic processes of the midpalatal suture.

Neural crest-derived mesenchymal cells proliferate, condense (Ost.b1), and directly differentiate into osteoblasts (Ost.b1, Ost.b2, and Ost.b3). Then, osteoblasts form an ossification center, secrete osteoid, and induce mineralization (Ost.b2). Osteoid is an unmineralized collagen–proteoglycan matrix that can bind calcium (Ost.b2). Subsequently, the osteoid will be surrounded by blood vessels and a trabecular/cancellous will form [39,40]. Furthermore, osteoblasts will be entrapped by osteoid and transform into osteocytes [41]. The bone remodeling occurs throughout the entire process (Ost.b3). We found that Ost.b3 expressed genes related to “osteoclast differentiation” (*Ctnnb1*, *Csf1*, *Mafb*, *Tgfb1*, *Tnfsf11*, *Fam20c*) using the R package gsfisher (GO:0030316, *p* value = 0.00418). Ost.b3 does not differentiate into osteoclasts but might interact with osteoclasts and promote osteoclastogenesis (Figure 6).

Intramembranous bones have been demonstrated to grow via remodeling, sutural growth, and secondary cartilages. Secondary cartilages give rise to the cartilage that rapidly differentiates into bone [42,43]. Similarly, we have found that Ost.b3 cells’ upregulated pathways are related to chondrocyte differentiation and cartilage development. Ost.b-9w are linked to cartilage development involved in endochondral bone morphogenesis. Additionally, some chondrogenic mesenchymal cells being mixed into the RNA velocity analysis suggests that chondrogenic mesenchymal cells might have a relationship with osteoblastic mesenchymal cells in terms of differentiation.

Then, we found that a type of *Piezo2^+^* chondrogenic mesenchymal cell might be associated with the detection of mechanical and external stimuli, indicating that these cells are potentially involved in osteogenic processes in the midpalatal suture. Most of the cells were from 4-week samples.

*Piezo2* is a rapidly adapting, evolutionarily conserved, mechanically activated ion channel gene that is mainly expressed in subsets of somatosensory neurons and Merkel cells [37]. *Piezo2* has been demonstrated to be essential for various mechanosensory responses, including sensing touch, proprioception, tactile pain, breathing, and blood pressure [44,45,46], and is essential for bone development and growth. Mechanical stimulation, including mechanical strain and muscle activity, have a significant influence on the shaping and growth of many intramembranous bones [42]. Furthermore, proprioception plays a vital role in the synchronization of bone formation, and the diminished input of proprioception could cause skeletal malformations [47]. In addition, *Piezo2* has been detected to be expressed directly in mouse bone stem cells that might be associated with mechanical signaling during bone formation and in chondrocytes that respond to constant mechanical loading [47,48,49].

In our study, *Piezo2^+^* chondrogenic mesenchymal cells upregulated pathways related to chondrocyte differentiation and mechanical stimulus response. Via mechanical stimulus detection, bones around the midpalatal suture could grow and remodel themselves into shapes adapted to mechanical strain. This is in accordance with our hypothesis egarding the unfused status of the midpalatal suture until adulthood: the mechanical stimulus of chewing induces the expansion of the midpalatal suture. *Piezo2^+^* cells might detect the expansion force and activate signaling factors in the osteogenesis process [50,51].

Similarly, Takahashi et al. reported that the midpalatal suture mainly consists of secondary cartilages and mesenchyme-like cells through immunohistochemical experiments. Type I collagen-rich and calcified bone matrix were found at the boundary between the precartilaginous and the cartilaginous cell layers after 14 days of orthodontic force application, suggesting that the expression of collagenous components might be altered by tensional force [33]. In our study, the pathways enriched in the *Piezo2^+^* chondrogenic mesenchymal cells are also related to fibroblast growth factor response, wound healing, and the differentiation of mesenchymal stem cells and chondrocytes. Therefore, we assume that these cells might play a role between pre-ossified and ossified bone surfaces. Our immunohistochemical staining results also identified the existence of *Piezo2^+^* chondrogenic mesenchymal cells inside the midpalatal suture and on the bilateral surfaces of the midpalatal suture. Their levels were highest at 4 weeks of age, less at 5 weeks of age, and lowest at 9 weeks of age.

Several studies have indicated some other mechano-sensation and mechano-transduction mechanisms in chondrocytes, including voltage-activated sodium and potassium channels and N-/L-type voltage-gated calcium channels [49,50,51]. Similarly, we found that *Piezo2^+^* chondrogenic mesenchymal cells show a higher expression of genes related to mechanical stimuli compared with *Piezo2^−^* ones, such as *Fos* and *Ier3*.

AP-1 is made up of members of the *Jun* (*Jun*, *Jun-B*, and *Jun-D*), *Fos* (*c-Fos*, *Fos-B*), and activating transcription factor (*ATF*) families.

These genes could be expressed in various cells to mediate the expression level of genes including growth factors, cytokines, and mechanical stress in response to many extracellular stimuli [52,53,54]. *Fos* is strongly expressed in osteocytes and on bone surfaces immediately (within 30 min) after the osteogenic stimulus [55], which is consistent with our hypothesis. Fluid shear-induced mechanical signaling in osteoblasts increases the expression of *Cox-2* and *c-Fos* within one hour [56]. *JunB* and *Fos* are differentially regulated by microenvironmental cues including chemical and mechanical signals and may work synergistically to regulate cell behaviors by regulating various transcription factors [57]. Moreover, *IER3* has been reported to be related to human mesenchymal stem cells’ response to intermittent shear stress [58]. Thus, the chondrogenic mesenchymal cells might assist osteoblastic mesenchymal cells in the process of intramembrane osteogenesis in the midpalatal suture, as well as in the detection of mechanical and external stimuli to remodel bone tissue [59]. Moreover, the normal intramembranous pathway in chicks includes a previously unrecognized transient chondrogenic phase similar to a prechondrogenic mesenchyme, and the cells in this phase retain chondrogenic potential that can be expressed in specific in vitro and in vivo microenvironments [60].

In a nutshell, our findings provide a thorough investigation of the midpalatal suture region with single-cell sequencing and assist in establishing a model of intramembrane osteogenesis in the midpalatal suture. Interestingly, we are the first to discover the existence of and put forward a hypothesis regarding the potential molecular signature of *Piezo2^+^* chondrogenic mesenchymal cells in this bone maturation and remodeling process.

## 5. Limitations of the Study

We first employed single-cell RNA sequencing to analyze intramembrane osteogenesis in the midpalatal suture region. There are still some limitations to our research. First, we only selected three time points for mice, which are equal to 5–6 years, 9–10 years, and 16–17 years in humans. These three timepoints include the key points of bone growth and development. In future, we will add more samples at different timepoints to clarify the sequential trend of intramembrane ossification. Second, mesenchyme cells were preliminarily analyzed in our study. We will further describe the midpalatal suture atlas over the time of growth. Third, we have proposed a hypothesis about the potential role of *Piezo2^+^* chondrogenic mesenchymal cells. This requires further experimental validation. Additionally, due to the limited accuracy of this operation, the sampling range is not narrowly focused on the tissue of the midpalatal suture, and thus the tissue cell scraping may partially damage its cyto-activity. The sampling process needs to be further optimized in future work.

## Data Availability

Requests for information or requests should be directed to the lead contact, Feng Chen (chenfeng2011@hsc.pku.edu.cn). This study did not generate new unique reagents.

## Supplementary Materials

The following supporting information can be downloaded at: https://www.mdpi.com/article/10.3390/cells11223585/s1. Figure S1. Quality control related to Figure 2.; Figure S2. Feature plots show the expression of genes characterizing nine subclusters of mesenchymal cells, related to Figure 2.; Figure S3. Expression of marker genes in mesenchymal cells, related to Figure 2.; Figure S4. Major cell types and mesenchyme cells subclusters at different time points, related to Figure 2.; Figure S5. Pseudotime trajectory of mesenchymal cells, related to Figure 2.; Figure S6. Gene expression associated with mechanical stimulus, related to Figure 4.; Figure S7. Identification of Piezo2+ fibroblastic mesenchymal cells, related to Figure 5.; Table S1. Cell number of major cell types in each cluster after batch correction, related to Figure 2.; Table S2. List of cluster-defining genes for mesenchymal cells, related to Figure 2.; Table S3. Cell number of each mesenchymal cluster cells after batch correction, related to Figure 2.; Table S4. List of gene ontology pathway information associated with pathways enriched in each cluster of osteoblastic mesenchymal cells divided by unsupervised clustering, related to Figure 3.; Table S5. List of gene ontology pathway information associated with pathways enriched in each cluster of osteoblastic mesenchymal cells divided by time, related to Figure 3.; Table S6. List of gene ontology pathway information associated with pathways enriched in chondrogenic mesenchymal cells, related to Figure 4.; Table S7. List of average expression level for the differentially expressed genes between Piezo2+ and Piezo2- chondrogenic mesenchymal cells, related to Figure 5. Table S8. List of gene ontology pathway information associated with pathways enriched in expression of Piezo2 in chondrogenic mesenchymal cells divided by different weeks of age, related to Figure 5.
