# Supplementary material for: Midpalatal Suture: Single-Cell RNA-Seq Reveals Intramembrane Ossification and Piezo2 Chondrogenic Mesenchymal Cell Involvement
Source: Cells. 2022 Nov 12;11(22):3585. doi: 10.3390/cells11223585 (PMC9688242; doi:10.3390/cells11223585)
Supplement: Supplementary file 1 [file cells-11-03585-s001.zip › cells-1974528-supplementary.pdf]

Supplementary Figures

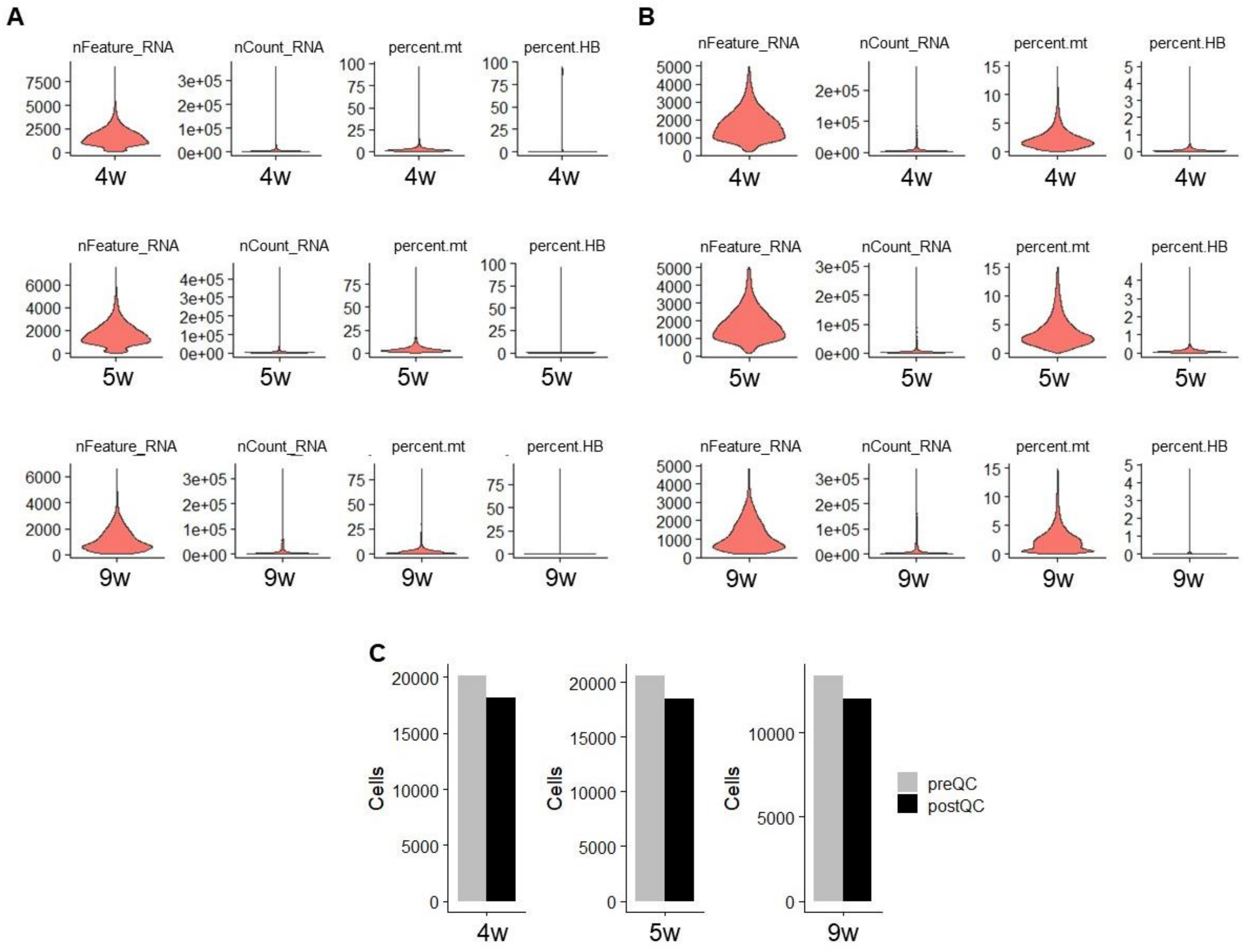

Figure S1. Quality control related to Figure 2. (A,B). Violin plots illustrating the number of features, RNA counts and percent mitochondrial transcripts from 4-week mice (4w), 5-week mice (5w), and 9-week mice (9w) samples prior to and after quality control. **(C).** Bar graphs showing the number of cells originally sequenced (pre-QC) and included for analysis (post-QC).

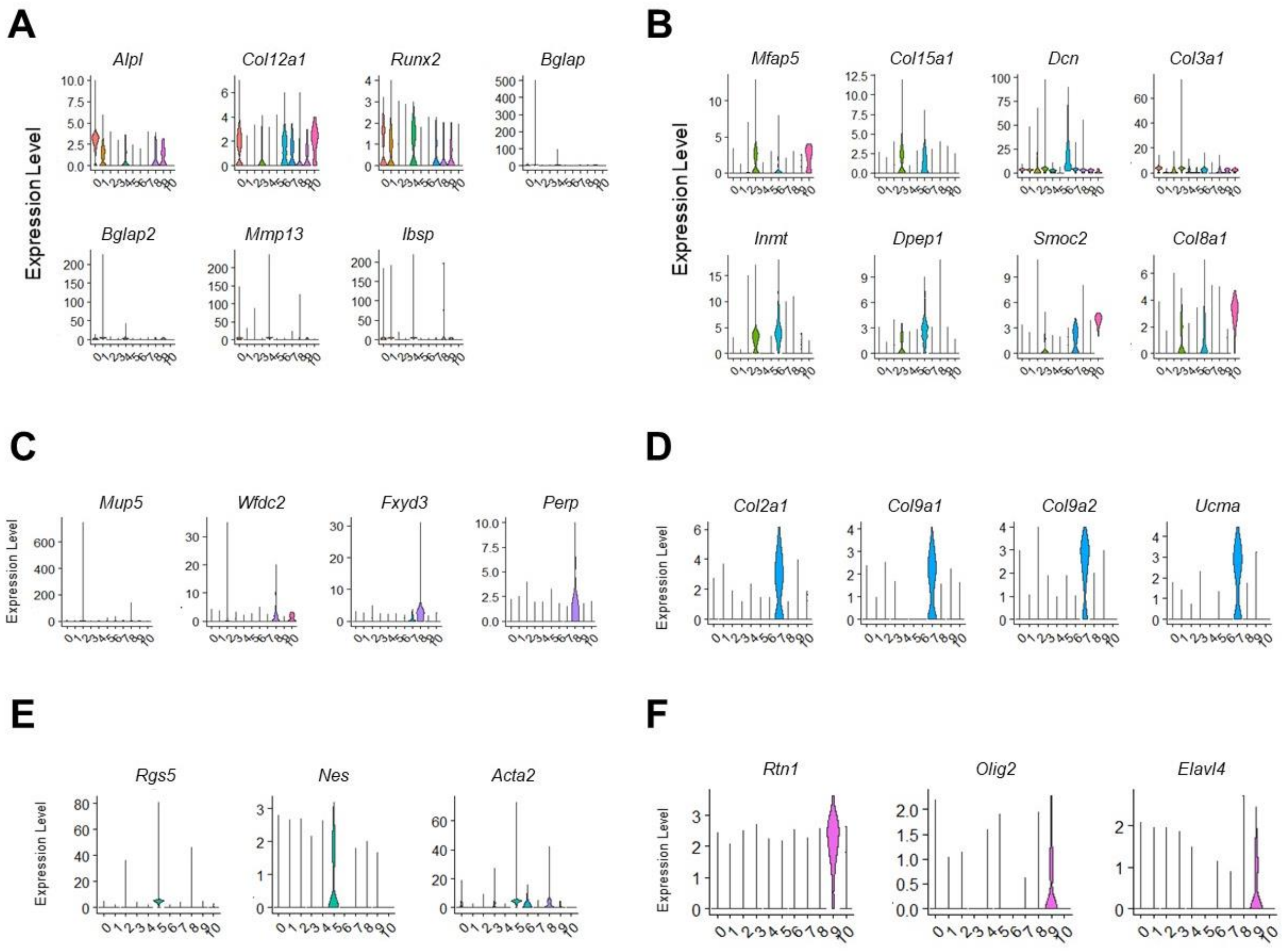

Figure S2. Feature plots show the expression of genes characterizing nine subclusters of mesenchymal cells, related to Figure 2. Eleven clusters were obtained from mesenchymal cells using unsupervised clustering and marker analysis. Three subclusters (cluster 0, 1, and 4) were identified within osteoblastic mesenchymal cells, expressing higher levels of *Alpl*, *Col2a1*, *Runx2*, *Bglap*, *Bglap2*, *Mmp13*, and *Ibsp* (Figure S2A). Three subclusters (cluster 3, 6 and 10) were identified within fibroblastic mesenchymal cells, showing higher expression of *Mfap5*, *Col15a1*, *Dcn*, *Col3a1*, *Inmt*, *Dpep1*, *Smoc2*, and *Col8a1* (Figure S2B). Two subclusters (cluster 2 and 8) were characterized by high expression of *Mup5*, *Wfdc2*, *Fxyd3*, and *Perp*, associated to secretory epithelial cells. These two subclusters were excluded in further analysis (Figure S2C). Cluster 7 was identified within chondrogenic mesenchymal cells with higher expression of *Mfap5*, *Col15a1*, *Dcn*, *Col3a1*, *Inmt*, *Dpep1*, *Smoc2*, and *Col8a1* (Figure S2D). Cluster 5 was marked by the expression of *Acta2*, *Rgs5*, and *Nes*, which has been proposed as a marker for pericytes (Figure S2E). *Rtn1*, *Olig2*, and *Elavl4* marked neural cells were found from Cluster 9 (Figure S2E). The aboved nine clusters were included in following analysis.

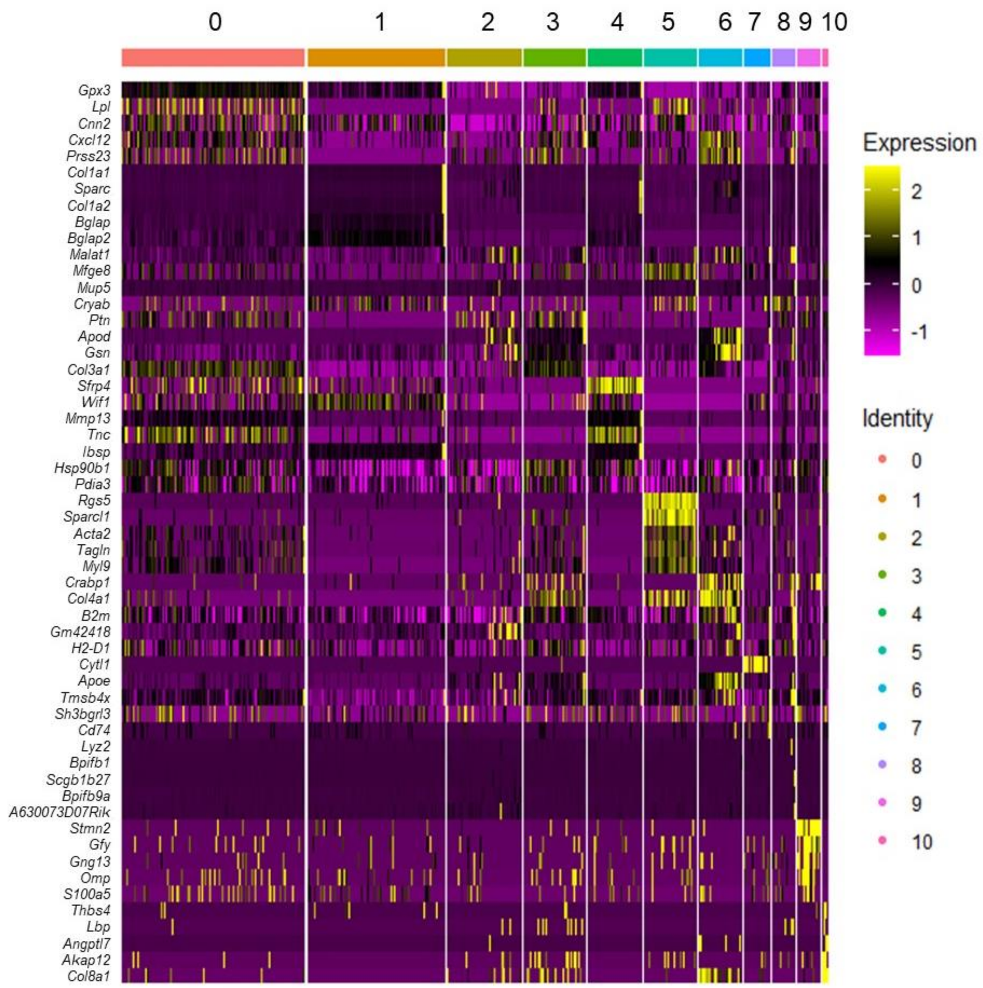

Figure S3. Expression of marker genes in mesenchymal cells, related to Figure 2. Heatmap show different expression level of marker genes in mesenchymal cells.

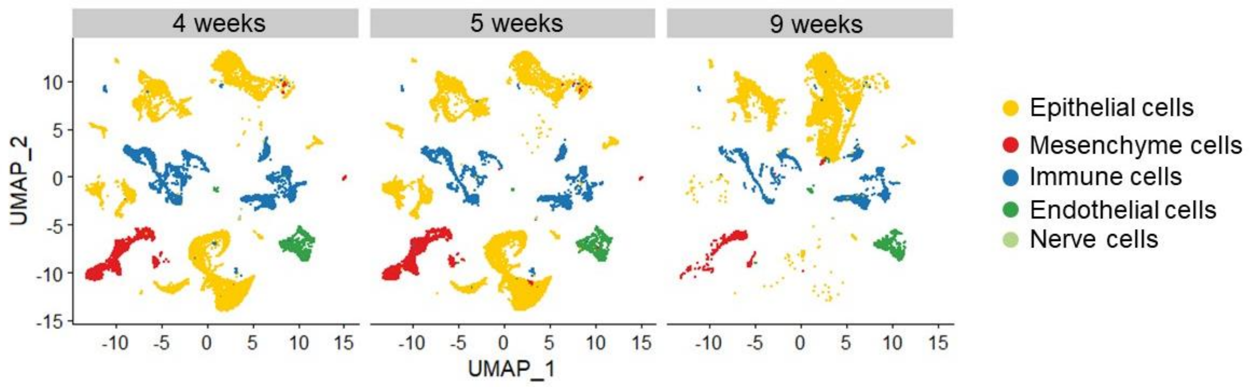

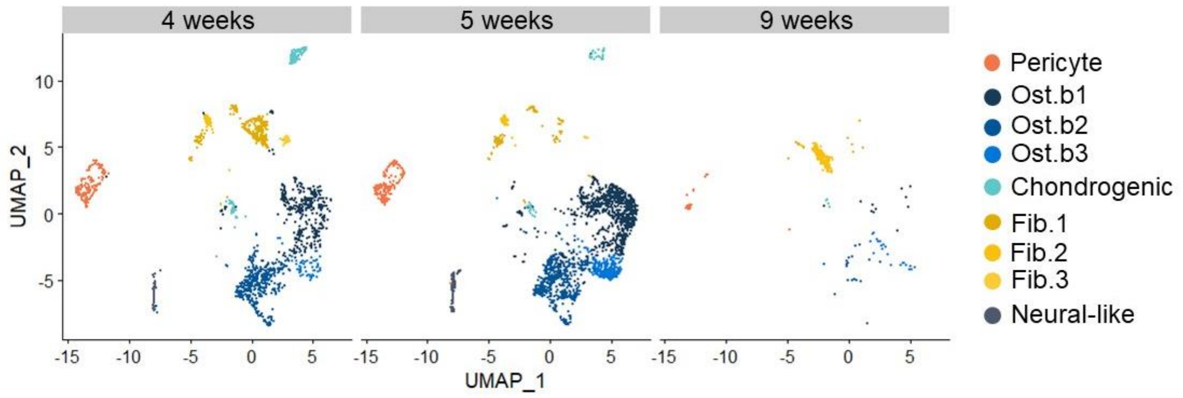

Figure S4. Major cell types and mesenchyme cells subclusters at different time points, related to Figure 2. (A). UMAP plots show distribution of five clusters, including epithelial cells, mesenchyme cells, immune cells, endothelial cells, and nerve cells, at 4 (16,686 cells), 5 (17,053 cells), and 9 (11,938 cells) weeks of age, respectively. **(B).** UMAP plots depict distribution of nine clusters from mesenchyme cells, at 4 (1,679 cells), 5 (2,435 cells), and 9 (564 cells) weeks of age, respectively.

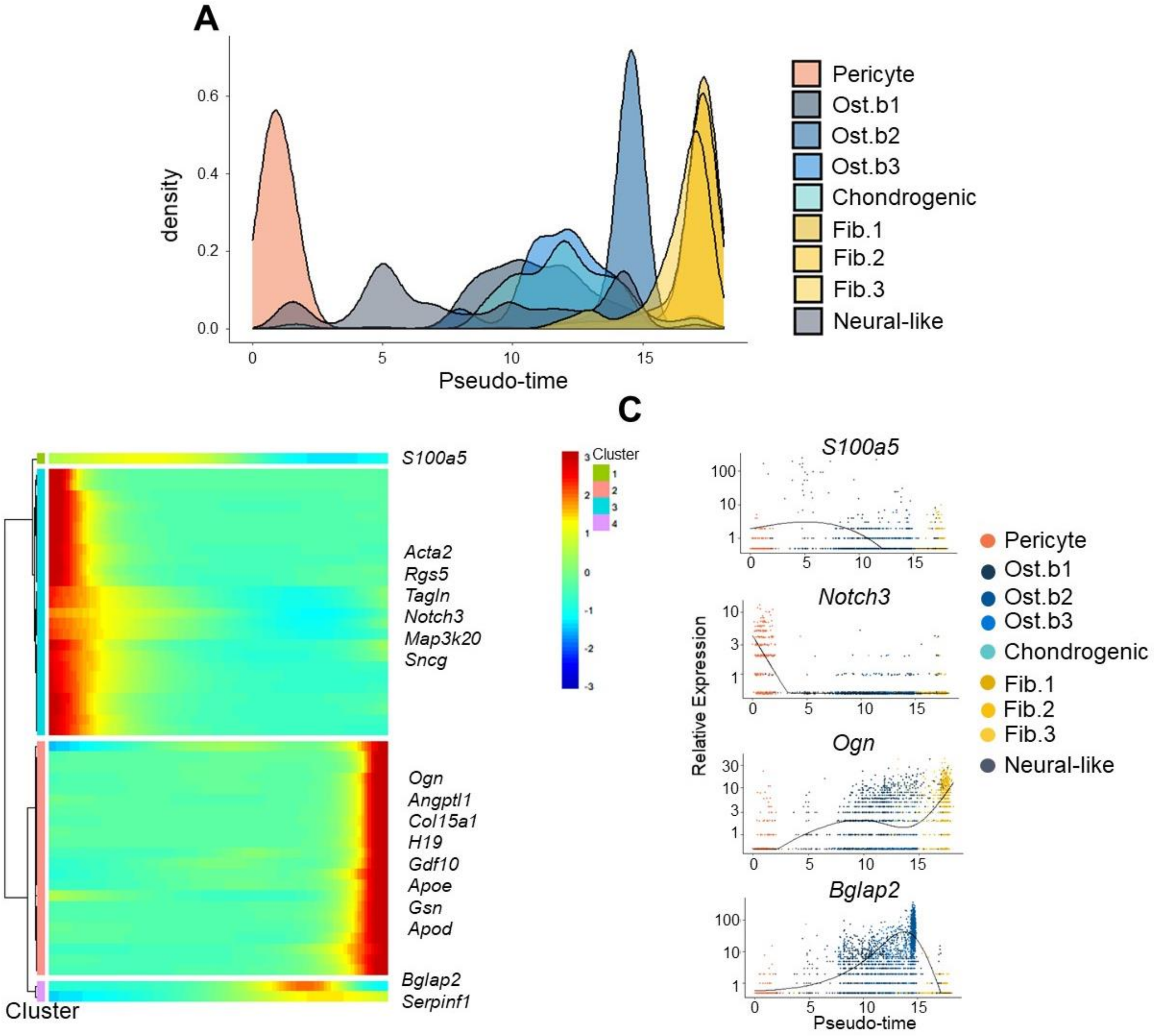

Figure S5. Pseudotime trajectory of mesenchymal cells, related to Figure 2. (A). The mesenchymal cell counts of each cluster along pseudo-time progression. **(B,C).** Heatmap show variation of gene expression level associated with mesenchymal cells pseudotime analysis. Specific genes including *S100a5*, *Notch3*, *Ogn*, and *Bglap2*, are listed on the right to depict the expression kinetics.

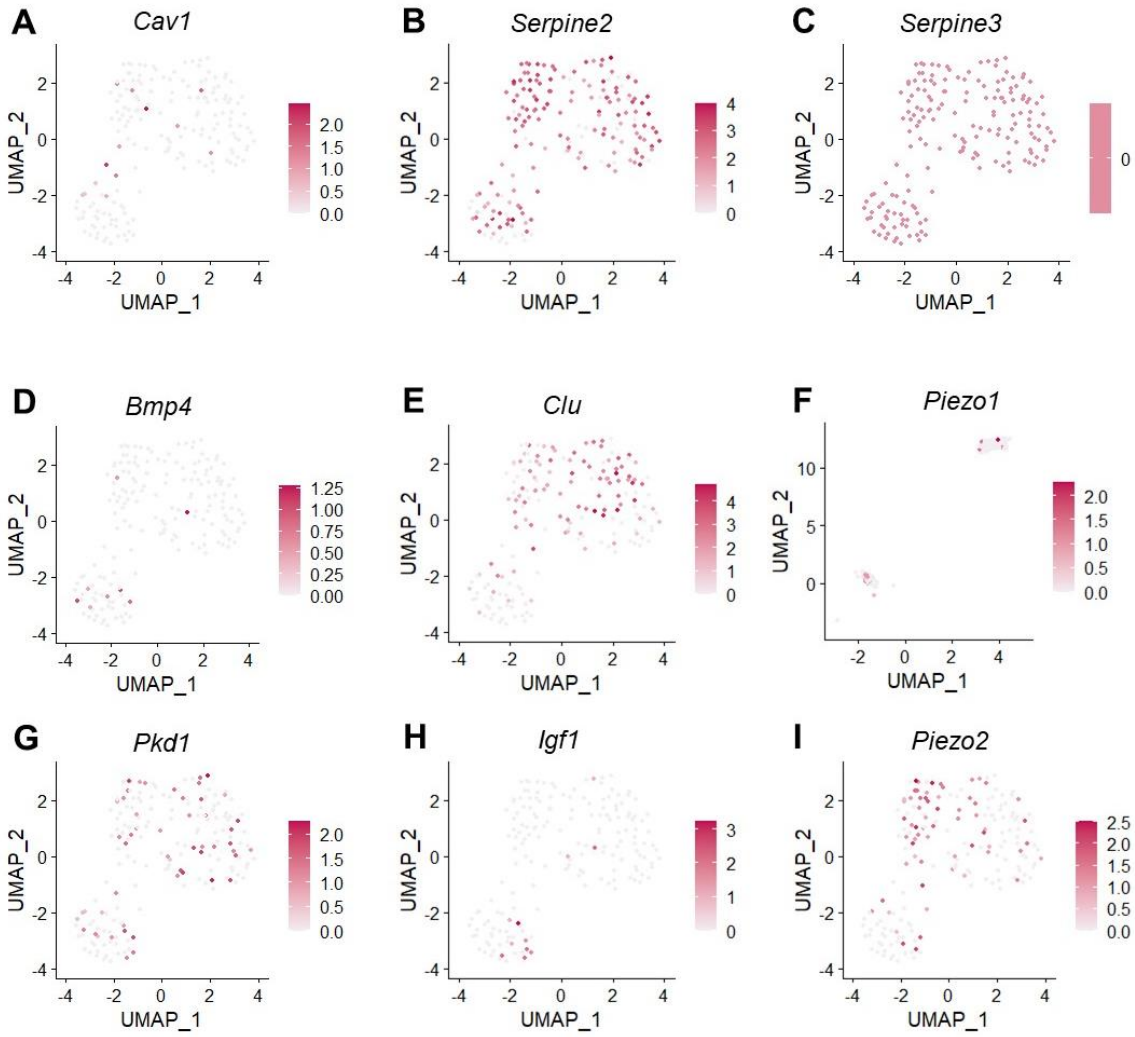

Figure S6. Gene expression associated with mechanical stimulus, related to Figure 4. UMAP representation of major cell types show the gene expression associated with mechanical stimulus, including *Cav1* (A), *Serpine2* (B), *Serpine3* (C), *Bmp4* (D), *Clu* (E), *Piezo1* (F), *Pkd1* (G), *Igf1* (H), and *Piezo2* (I). Color coded by the expression level of each gene above.

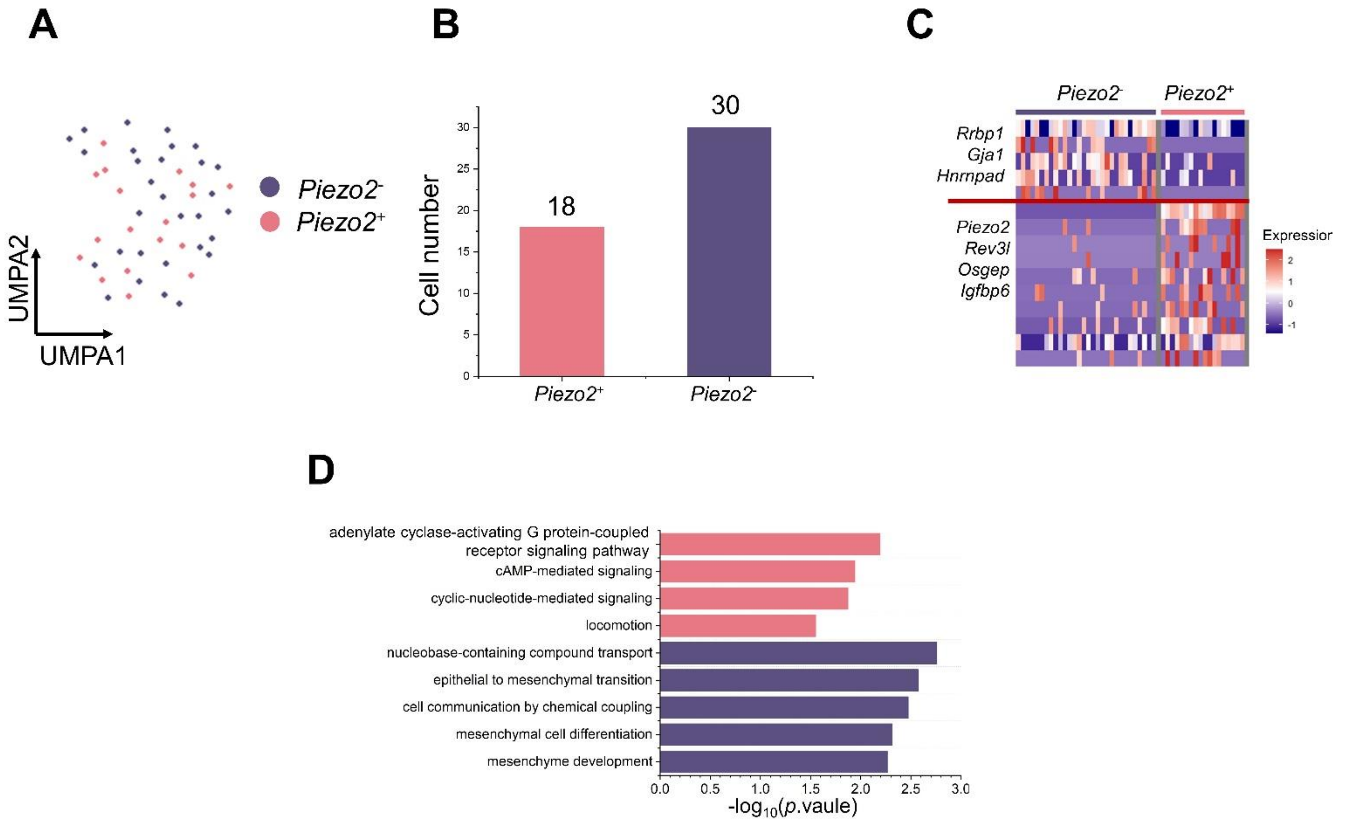

Figure S7. Identification of *Piezo2*$^{+}$ fibroblastic mesenchymal cells, related to Figure 5. (A). Feature plots show the expression of *Piezo2*$^{+}$ and *Piezo2*$^{-}$ fibroblastic mesenchymal cells. Color coded by whether the cells expressed *Piezo2*. (B). Bar graphs show number of *Piezo2*$^{+}$ (18 cells) and *Piezo2*$^{-}$ (30 cells) fibroblastic mesenchymal cells. (C). Heatmap show average expression level of the top differentially expressed genes in *Piezo2*$^{+}$ and *Piezo2*$^{-}$ fibroblastic mesenchymal cells. (D). Bar graphs show enriched gene ontology terms of *Piezo2*$^{+}$ and *Piezo2*$^{-}$ fibroblastic mesenchymal cells.

Supplementary Tables

Table S1. Cell number of major cell types in each cluster after batch correction, related to Figure 2.

Cell type	4-week	5-week	9-week
Mesenchymal cell	1679	2435	564
Endothelial cells	872	744	609
Epithelial cells	8729	11938	8925
Immune cells	5317	1910	1836
Nerve cells	89	26	4

Table S2. List of cluster-defining genes for mesenchymal cells, related to Figure 2.

Gene	p_val	avg_log2FC	pct.1	pct.2	p_val_adj	Cluster
Alpl	0	3.879965	0.901	0.3	0	Ost.b1
Lum	2.87E-226	1.65069	0.926	0.617	1.03E-221	Ost.b1
Col12a1	5.19E-210	1.496159	0.676	0.229	1.86E-205	Ost.b1
Runx2	8.82E-47	0.776044	0.477	0.304	3.16E-42	Ost.b1
Col1a1	0	866.1659	1	0.873	0	Ost.b2
Col1a2	0	618.0319	1	0.891	0	Ost.b2
Bglap	0	589.1684	0.979	0.437	0	Ost.b2
Bglap2	0	267.4474	0.972	0.397	0	Ost.b2
Mfap5	1.83E-200	8.404545	0.515	0.06	6.57E-196	Fib.1
Col4a5	1.42E-149	1.269414	0.299	0.047	5.11E-145	Fib.1
Col8a1	2.16E-87	1.120717	0.404	0.101	7.76E-83	Fib.1
Col6a2	3.01E-80	1.294612	0.722	0.398	1.08E-75	Fib.1
Dcn	1.40E-76	14.87951	0.898	0.81	5.02E-72	Fib.1
Mmp13	2.82E-74	133.3894	0.989	0.373	1.01E-69	Ost.b3
Ibsp	8.80E-63	36.7156	0.997	0.472	3.16E-58	Ost.b3
Runx2	2.54E-61	0.675788	0.571	0.331	9.12E-57	Ost.b3
Rgs5	8.51E-38	54.05698	0.921	0.08	3.05E-33	Pericyte
Sparcl1	6.97E-214	49.18616	0.989	0.127	2.50E-209	Pericyte
Acta2	1.81E-137	48.28633	0.921	0.249	6.48E-133	Pericyte
Tagln	4.77E-26	78.57289	0.89	0.241	1.71E-21	Pericyte
Myl9	0	72.59973	0.937	0.348	0	Pericyte
Inmt	0	6.25153	0.956	0.111	0	Fib.2
Dpep1	2.10E-226	2.78564	0.901	0.098	7.52E-222	Fib.2
Mfap4	1.57E-219	6.87238	0.946	0.181	5.63E-215	Fib.2
Crabp1	1.59E-200	19.63212	0.759	0.106	5.69E-196	Fib.2
Col18a1	0	2.852967	0.571	0.096	0	Fib.2
Col4a2	0	7.334275	0.755	0.193	0	Fib.2
Col2a1	2.91E-228	5.311917	0.701	0.023	1.04E-223	Chondrogenic
Ucma	1.67E-223	3.966211	0.701	0.003	6.00E-219	Chondrogenic
Col9a2	1.81E-128	3.628241	0.768	0.036	6.51E-124	Chondrogenic
Col9a3	3.25E-128	3.186478	0.706	0.009	1.16E-123	Chondrogenic
Col9a1	0	3.111737	0.678	0.005	0	Chondrogenic
Piezo2	0	0.91682	0.35	0.037	0	Chondrogenic
Rtn1	0	3.154456	0.916	0.055	0	Neural-like
Stmn3	0	2.437515	0.839	0.032	0	Neural-like
Elavl3	0	2.202234	0.845	0.027	0	Neural-like
Stmn2	1.83E-78	3.837181	0.91	0.073	6.57E-74	Neural-like
Olig2	0	0.816125	0.368	0.005	0	Neural-like
Elavl4	0	0.946846	0.374	0.01	0	Neural-like
Col8a1	0	3.432562	0.938	0.119	0	Fib.3
Smoc2	3.13E-286	1.221796	1	0.179	1.12E-281	Fib.3
Piezo2	1.27E-252	1.588681	0.375	0.046	4.56E-248	Fib.3
Col12a1	2.68E-198	1.432669	0.75	0.343	9.63E-194	Fib.3

Table S3. Cell number of each mesenchymal cluster cells after batch correction, related to Figure 2.

Cell type	4-week	5-week	9-week
Pericyte	162	179	24
Ost.b1	365	866	11
Ost.b2	393	529	18
Ost.b3	61	288	22
Chondrogenic	127	46	4
Fib.1	317	91	13
Fib.2	74	32	188
Fib.3	44	4	0
Neural-like	37	118	0

Table S4. List of gene ontology pathway information associated with pathways enriched in each cluster of osteoblastic mesenchymal cells divided by unsupervised clustering, related to Figure 3.

geneset_id	Description	fg_freq	bg_freq	n_fg	n_bg	n_set	p.val	Cluster	p.adj
GO:0001570	Vasculogenesis	0.022082	0.007105	7	16	110	0.003748	Ost.b1	0.061752
GO:0045667	Regulation of osteoblast differentiation	0.041009	0.018206	13	41	173	0.002854	Ost.b1	0.053734
GO:0060485	Mesenchyme development	0.059937	0.030639	19	69	364	0.002189	Ost.b1	0.05156
GO:0009612	Response to mechanical stimulus	0.031546	0.011101	10	25	192	0.001218	Ost.b1	0.040589
GO:0001957	Intramembranous ossification	0.0059	0.000889	2	2	5	0.022644	Ost.b2	0.121761
GO:0001649	Osteoblast differentiation	0.050147	0.028	17	63	266	0.009375	Ost.b2	0.092107
GO:0110148	Biomineralization	0.044248	0.02	15	45	215	0.001578	Ost.b2	0.045905
GO:0030198	Extracellular matrix organization	0.070796	0.032889	24	74	386	0.000107	Ost.b2	0.008886
GO:0030316	Osteoclast differentiation	0.04	0.009641	5	22	158	0.005641	Ost.b3	0.073655
GO:0051216	Cartilage development	0.08	0.020158	10	46	286	0.000128	Ost.b3	0.009632
GO:0048771	Tissue remodeling	0.08	0.018843	10	43	229	6.95E-05	Ost.b3	0.00711
GO:0110148	Biomineralization	0.088	0.021034	11	48	215	3.43E-05	Ost.b3	0.004225

Table S5. List of gene ontology pathway information associated with pathways enriched in each cluster of osteoblastic mesenchymal cells divided by time, related to Figure 3.

geneset_id	Description	fg_freq	bg_freq	n_fg	n_bg	n_set	p.val	Cluster	p.adj
GO:0030198	Extracellular matrix organization	0.077519	0.037975	10	60	386	0.02045	Ost.b-4w	0.118263
GO:0010464	Regulation of mesenchymal cell proliferation	0.03876	0.007595	5	12	50	0.001666	Ost.b-4w	0.046749
GO:0010463	Mesenchymal cell proliferation	0.03876	0.007595	5	12	67	0.001666	Ost.b-4w	0.046749
GO:0001649	Osteoblast differentiation	0.1	0.031654	4	49	266	0.035034	Ost.b-5w	0.151827
GO:0070661	Leukocyte proliferation	0.1	0.017442	4	27	448	0.00437	Ost.b-5w	0.063727
GO:0030316	Osteoclast differentiation	0.075	0.008398	3	13	158	0.003823	Ost.b-5w	0.059714
GO:0060351	Cartilage development involved in endochondral bone morphogenesis	0.04	0.005161	2	8	43	0.025224	Ost.b-9w	0.129711
GO:0009612	Response to mechanical stimulus	0.06	0.011613	3	18	192	0.018321	Ost.b-9w	0.112141
GO:0110148	Biomineralization	0.1	0.021935	5	34	215	0.003911	Ost.b-9w	0.059714

Table S6. List of gene ontology pathway information associated with pathways enriched in chondrogenic mesenchymal cells, related to Figure 4.

geneset_id	Description	fg_freq	bg_freq	n_fg	n_bg	n_set	p.val	p.adj
GO:0050906	Detection of stimulus involved in sensory perception	0.010989	0.003135	3	5	188	0.037901	0.241305
GO:0050982	Detection of mechanical stimulus	0.010989	0.003135	3	5	73	0.037901	0.241305
GO:0009581	Detection of external stimulus	0.014652	0.004389	4	7	160	0.019126	0.200977
GO:0009582	Detection of abiotic stimulus	0.014652	0.004389	4	7	159	0.019126	0.200977
GO:0030198	Extracellular matrix organization	0.095238	0.048276	26	77	386	0.000208	0.026823
GO:0002062	Chondrocyte differentiation	0.054945	0.020063	15	32	147	7.72E-05	0.013907
GO:0098743	Cell aggregation	0.032967	0.006897	9	11	30	4.49E-06	0.002621
GO:0051216	Cartilage development	0.084249	0.032602	23	52	286	3.03E-06	0.002118
GO:0061448	Connective tissue development	0.095238	0.039498	26	63	374	3.3E-06	0.002118
GO:0001502	Cartilage condensation	0.032967	0.005643	9	9	24	1.13E-07	0.000181

Table S7. List of average expression level for the differentially expressed genes between *Piezo2*$^{+}$ and *Piezo2*$^{-}$ chondrogenic mesenchymal cells, related to Figure 5.

	p_val	avg_log$_2$FC	pct.1	pct.2	p_val_adj	Cluster
Dcn	7.25E-06	3.436791	0.896	0.952	0.260127	*Piezo2*$^{-}$
Mgp	2.52E-05	46.71951	0.852	1	0.903961	*Piezo2*$^{-}$
Ibsp	0.002264	2.705144	0.348	0.161	1	*Piezo2*$^{-}$
Lum	0.005311	5.807815	0.583	0.468	1	*Piezo2*$^{-}$
Col1a1	0.006119	4.559843	0.635	0.548	1	*Piezo2*$^{-}$
Piezo2	7E-38	2.140728	1	0	2.51E-33	*Piezo2*$^{+}$
Junb	6.5E-09	4.088985	0.952	0.878	0.000233	*Piezo2*$^{+}$
Ier3	0.000317	13.31389	0.855	0.652	1	*Piezo2*$^{+}$
Fos	0.002001	2.847994	0.968	0.87	1	*Piezo2*$^{+}$

Table S8. List of gene ontology pathway information associated with pathways enriched in expression of *Piezo2* in chondrogenic mesenchymal cells divided by different weeks of age, related to Figure 5.

geneset_id	Description	fg_freq	bg_freq	n_fg	n_bg	n_set	p.val	Cluster	p.adj
GO:1902548	Negative regulation of cellular response to vascular endothelial growth factor stimulus	1	0.000562	1	1	11	0.000562	*Piezo2*$^{-}$	0.064389
GO:0019800	Peptide cross-linking via chondroitin 4-sulfate glycosaminoglycan	1	0.001124	1	2	9	0.001124	*Piezo2*$^{-}$	0.064389
GO:0030204	Chondroitin sulfate metabolic process	1	0.003371	1	6	41	0.003371	*Piezo2*$^{-}$	0.064389
GO:0090303	Positive regulation of wound healing	0.1875	0.007299	3	13	68	0.000161	*Piezo2*$^{+}$	0.064389
GO:0071774	Response to fibroblast growth factor	0.1875	0.010107	3	18	144	0.000448	*Piezo2*$^{+}$	0.064389
GO:0044330	Canonical Wnt signaling pathway involved in positive regulation of wound healing	0.0625	0.000561	1	1	1	0.008984	*Piezo2*$^{+}$	0.064389
GO:0098759	Cellular response to interleukin-8	0.0625	0.000561	1	1	2	0.008984	*Piezo2*$^{+}$	0.064389
GO:0009612	Response to mechanical stimulus	0.125	0.011791	2	21	192	0.014389	*Piezo2*$^{+}$	0.076585
GO:0032332	Positive regulation of chondrocyte differentiation	0.0625	0.002246	1	4	31	0.035483	*Piezo2*$^{+}$	0.090909
GO:0061036	Positive regulation of cartilage development	0.0625	0.002246	1	4	43	0.035483	*Piezo2*$^{+}$	0.090909
GO:2000741	Positive regulation of mesenchymal stem cell differentiation	0.0625	0.002246	1	4	4	0.035483	*Piezo2*$^{+}$	0.090909
GO:0104004	Cellular response to environmental stimulus	0.125	0.021336	2	38	330	0.044077	*Piezo2*$^{+}$	0.097502
